# Congenital Lumbar Hernia with Lumbocostovertebral Syndrome: A Case Report and Review of the Literature

**DOI:** 10.1155/2013/532910

**Published:** 2013-09-18

**Authors:** Ketan Vagholkar, Khojasteh Dastoor

**Affiliations:** ^1^Department of Surgery, Dr. D. Y. Patil Medical College, Navi Mumbai, Maharashtra 400706, India; ^2^Annapurna Niwas, 229 Ghantali Road, Thane 400602, India

## Abstract

*Introduction*. Congenital lumbar hernia is one of the rare types of hernias. Anomalies of the ribs, spine, and muscles which constitute the lumbocostovertebral syndrome in association with congenital lumbar hernia make it the rarest of entities. In addition, a multitude of other organ systems may be involved. *Case Report*. A case of congenital lumbar hernia associated with lumbocostovertebral syndrome is presented in view of its rarity and diagnostic and therapeutic challenges. *Discussion*. Anatomical background of congenital lumbar hernia associated with various other anomalies especially of the musculoskeletal structures is discussed. All cases of congenital lumbar hernia should be investigated for other congenital anomalies. Both open and laparoscopic approaches have been described for surgical treatment. *Conclusion*. Open surgical intervention is the mainstay of treatment taking into consideration the technical challenges posed by distorted anatomy due to the associated congenital anomalies.

## 1. Introduction

Congenital hernias in the lumbar region are a rarity. They account for only 10% of all the lumbar hernias. Congenital lumbar hernia is associated with a multitude of congenital anomalies involving various other organ systems of the body. These involve the ribs, spine, muscles, kidneys, and spinal meninges. Less than 50 cases of congenital lumbar hernia associated with other congenital anomalies have been reported in English literature making it a rare entity [1, 2]. A case of congenital lumbar hernia associated with lumbocostovertebral syndrome is reported along with a review of the literature.

## 2. Case Report

**Figure 1:**
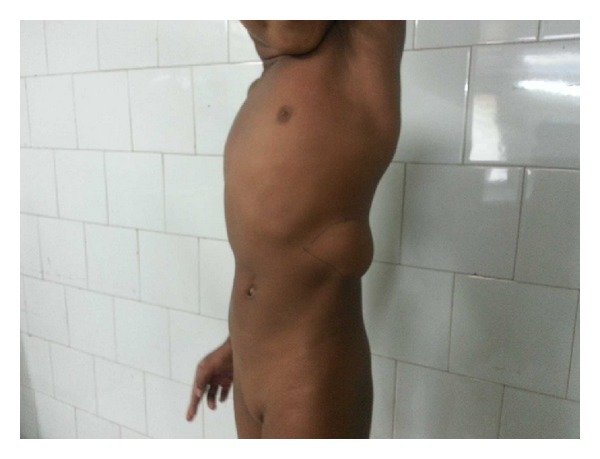
Left-sided congenital lumbar hernia.

**Figure 2:**
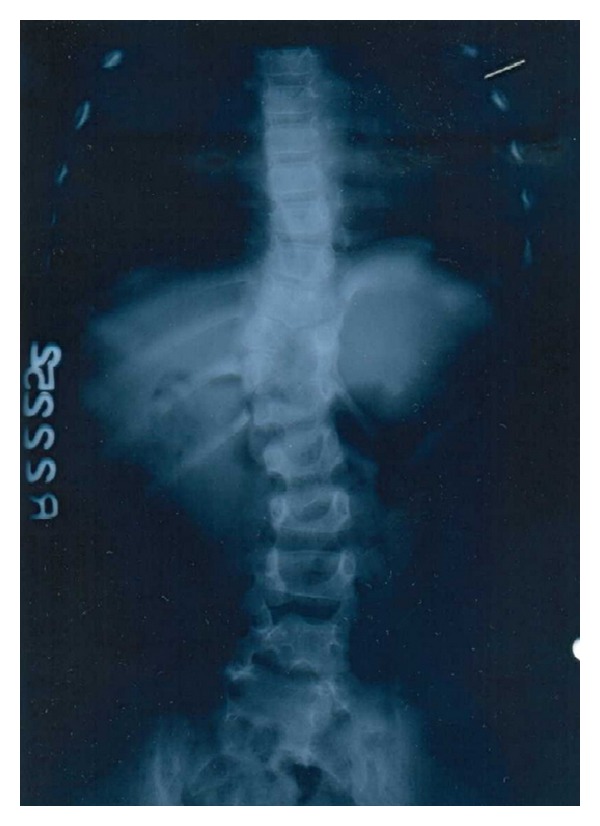
X-ray of the chest region and abdomen showing absence of lower ribs on the left side and scoliosis.

**Figure 3:**
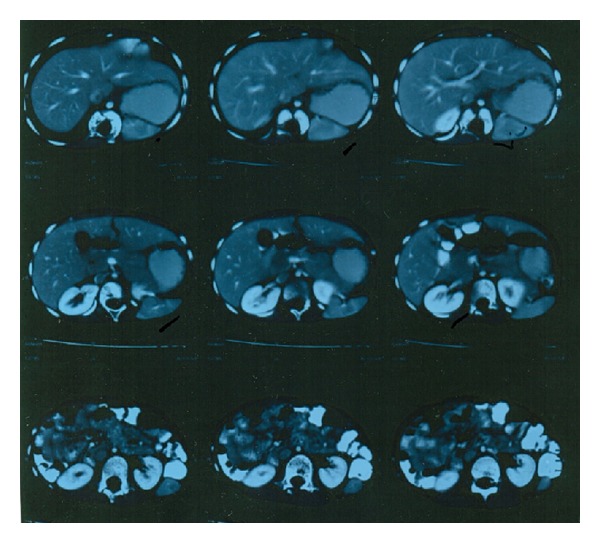
CT scan showing severely hypoplastic posterior abdominal muscles (erector spinae, iliocostalis, and latissimus dorsi) causing posterolateral herniation of the spleen. D10–12 vertebral bodies are hemivertebrae.

A 5-year-old girl presented with a swelling arising from the lateral abdominal wall on the left side. As per the mother's description the swelling was present since birth and had increased in size over a period of time (Figure 1). The girl had no other symptoms related to the digestive and urinary system. On physical examination the left posterolateral abdominal wall swelling had a visible and palpable expansile impulse on coughing with the defect size measuring approximately 4 cms in diameter. Hematological investigations did not reveal any abnormality. Chest X-ray revealed the absence of the lower ribs on the left side with scoliosis of the spine (Figure 2). A contrast-enhanced CT scan of the abdomen revealed quite a few abnormalities. They were absence of the lower two ribs on the left side, attenuation of the left posterolateral body wall musculature (erector spinae, iliocostalis, and latissimus dorsi), a musculoaponeurotic defect with herniation of the spleen, hemivertebrae, and scoliosis (Figure 3). The renal system was normal.

**Figure 4:**
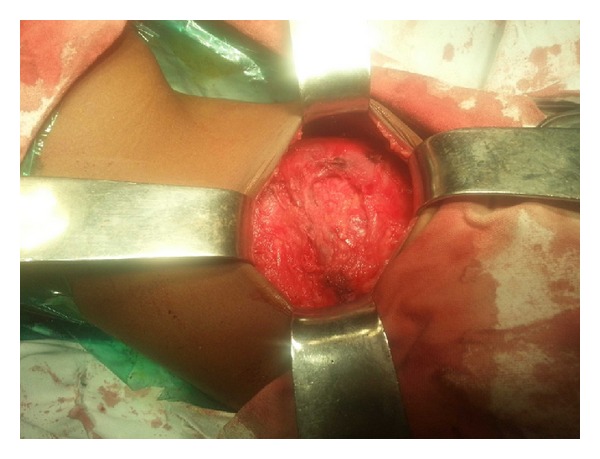
The defect in the hypoplastic musculature.

**Figure 5:**
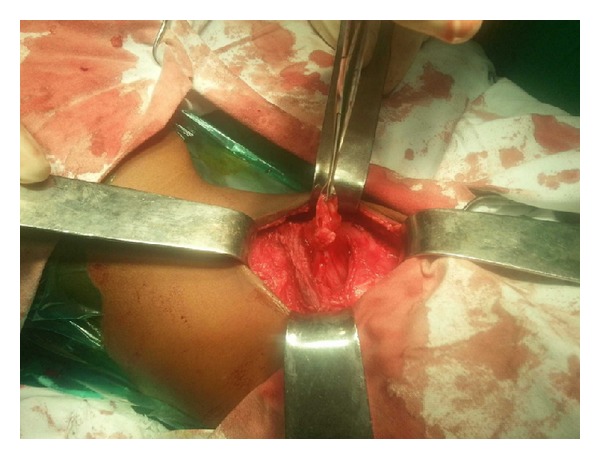
Defect in the attenuated musculature with the sac dissected up to the neck.

**Figure 6:**
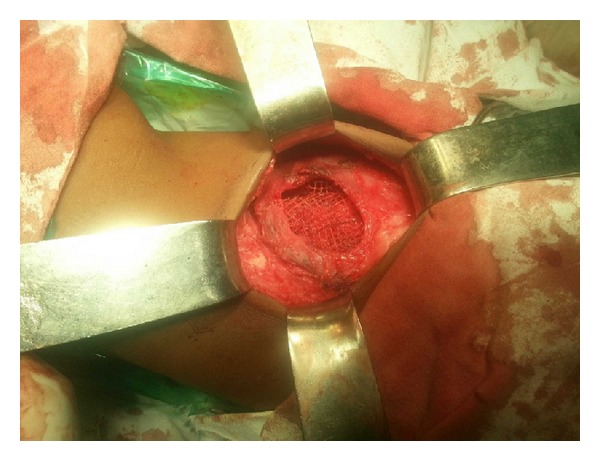
A prosthetic mesh placed between the inverted sac and the muscular layer.

**Figure 7:**
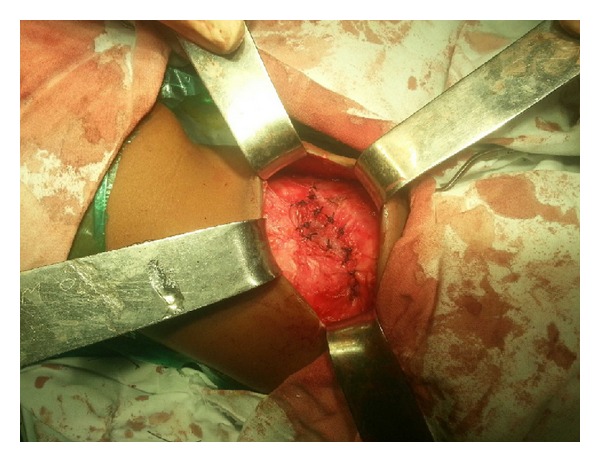
The defect closed over the mesh with overlapping flaps of the adjacent musculature.

The patient underwent open surgery for repair of the hernia. The defect was identified amidst hypoplastic surrounding musculature (Figure 4). The sac was identified and dissected all around up to the neck (Figure 5). The sac was pushed in by means of a circumferential purse string suture. A semiabsorbable prosthetic mesh was placed over the defect with overlapping flap closure of the adjacent musculature (Figures 6 and 7). Postoperative recovery was uneventful. Patient was discharged on the fourth postoperative day. The patient has been following up for 6 months without any recurrence.

## 3. Discussion

The lumbar region is defined anatomically by the 12th rib superiorly, erector spinae muscles medially, crest of the iliac bone inferiorly, and the external oblique muscle laterally. This space is further divided into superior lumbar triangle of Grynfeltt-Lesshaft and inferior lumbar triangle of Petit [3]. The superior lumbar triangle is an inverted space bordered at the base by the 12th rib and at the lower edge of the serratus posterior inferior muscle, posteriorly by the sacrospinalis muscle and interiorly by the internal oblique muscle. The floor consists of the transversalis fascia and the aponeurosis of the transversus muscle of the abdomen. The inferior lumbar triangle of Petit is smaller and is bordered by the crest of the iliac bone at the base, latissimus dorsi medially, and the external oblique muscle laterally. The floor is formed by the lumbodorsal fascia adjacent to the aponeurosis of the internal oblique and transversus muscles.

Predisposition to herniation in the superior triangle depends upon a multitude of factors such as size and forms of the triangle, length and angulation of the rib, size of the quadratus lumborum and serratus posterior muscles, the insertion of the latissimus dorsi between the 11th and 12th ribs, variable insertion of the external oblique above the 12th rib, and whether the internal oblique is muscular or aponeurotic at its insertion above the 12th rib [3].

The muscular attachments of the 11th and 12th ribs have a significant impact on strengthening the region. With the absence of the 11th & 12th ribs the otherwise involved adjacent musculature is attenuated with weakened attachments. This predisposes to congenital lumbar hernia in cases associated with lumbocostovertebral syndrome [4]. Approximately 10% of all lumbar hernias are congenital, and the vast majority is unilateral. The majority of patients present early usually in the first or second year of life as in the case presented.

A variety of anomalies of the musculoskeletal system are observed in these patients. These include absent ribs, hemivertebrae, and posterior spinal dysraphism. These constitute the entity: lumbocostovertebral syndrome [5, 6]. A myelomeningocele or intrathoracic neuroblastomas have also been reported [7]. Amongst the urogenital anomalies pelviureteric junction obstruction and cloacal extrophy have been reported [8]. Concomitant diaphragmatic hernia has also been reported [8]. Other orthopedic anomalies such as congenital club foot and arthrogryposis have been reported [9]. Atrial septic defects in rare instances have also been reported [9]. Therefore a patient diagnosed as having congenital lumbar hernia requires an extensive workup to detect and diagnose various other congenital anomalies [9]. 

Contrast-enhanced CT scan of the abdomen along with plain radiology helps in detecting all the associated anomalies of the other organ systems.

 Surgery remains the mainstay of treatment [10, 11]. Though there are anecdotal case reports suggesting laparoscopic approach, the technical advantages of the open method still outweigh the laparoscopic method [12].

An open approach is preferable in view of the anticipated fact that the local anatomy is distorted with a variety of variations rendering anatomical identification of structures as difficult. Precise definition of the defect, followed by identification of the surrounding muscles, is a prerequisite for successful outcome. The sac needs to be identified and dissected up to the neck. The sac need not be opened as the hernia comprises of an extra peritoneal protrusion of the organs such as the spleen or at times the colon. In the case presented the inferior pole of the spleen was the content of the sac [13]. Proper head low repositioning of the patient enabled reduction of the sac and its contents. The surrounding muscles were dissected,undermined and flaps were created. A purse string suture taken all around the neck of the sac and tightened ensures reduction. A semiabsorbable mesh comprising polypropylene and polyglactin is used so that the growth potential of the already-distorted area is not hampered. The mesh is fixed to the overhanging attenuated muscles with nonabsorbable suture material. Flaps created from the available surrounding musculature are then approximated.

The other advantage of an open method is that utmost care can be exercised when dealing with the sac. Inadvertent opening of the sac could lead to damage to the underlying structures. Creating a working space for laparoscopic intervention poses the greatest challenge in congenital lumbar hernia. This could involve damage to the contents. Placement and fixation of the mesh may also be technically difficult due to attenuated musculature leading to failure of the surgery.

## 4. Conclusion

Congenital lumbar hernia with lumbocostovertebral syndrome is one of the rare types of hernias found in pediatric age group. Diagnosis of congenital hernia should alert the surgeon, prompting further investigations for various other congenital orthopedic, neurological, and urological anomalies. Open prosthetic repair continues to be the procedure of choice for treating such hernias.
